# Regulatory effects on particulate pollution in the early hours of Chinese New Year, 2015

**DOI:** 10.1007/s10661-017-6167-0

**Published:** 2017-08-23

**Authors:** Yonghang Lai, Peter Brimblecombe

**Affiliations:** School of Energy and Environment, City University of Hong Kong, Kowloon Tong, Hong Kong

**Keywords:** China, Fireworks, Hong Kong, PM2.5, PM10, Taiwan

## Abstract

Human activities are a key driver of air pollution, so it is hardly surprising that celebrations affect air quality. The use of fireworks contributes to high particulate concentrations in many parts of the world, with the Chinese Lunar New Year (spring festival) particularly noticeable, as firecrackers are traditionally used to drive off evil spirits. Fireworks lead to short-term peaks in the concentration of PM10, PM2.5 and SO_2_. Regulatory actions that restrict the use of fireworks have been evident in China since the 1990s. This paper investigates the particulate concentrations in nine Chinese cities (Beijing, Chengdu, Chongqing, Tianjin, Xi’an, Nanjing, Shanghai, Guangzhou and Shenzhen, along with Hong Kong (a Special Administrative Region) and Taipei and Kaohsiung (Taiwan) with a particular focus on the celebrations of 2015. Extremely high concentrations of particulate matter were observed, with some sites revealing peak PM10 concentrations in excess of 1000 μg m^−3^ in the early hours of the New Year. In Beijing, Tianjin and Chongqing, the activities caused high particulate matter concentrations at most sites throughout the city. These peaks in particulate load in the early hours of Chinese New Year do not appear to be closely related to meteorological parameters. However, in cities where fireworks appear to be better regulated, there are fewer sharp pollution peaks just after midnight, although lowered air quality can still be found in the outer parts of some cities, remote from regulatory pressures. A few cities seem to have been effective at reducing the impact of the celebrations on air quality, with Nanjing a recent example. An increasing focus on light displays and electric lanterns also seems to offer a sense of celebration with much reduced impacts on air quality.

## Introduction

Human activities are a key driver of air pollution so it is hardly surprising that celebrations and cultural activities can have a profound impact. One of the best studied is the regularly occurring weekend effect, which is defined as the difference of air pollutant concentrations between weekdays and weekends, with a general reduction of major pollutants. For instance, Qin et al. (2004) reported that the average concentrations of NOx, CO, NMOC and PM10 at weekends were lower than those on weekdays in southern California. It was first observed in the USA in the 1970s (Jimenez et al. 2005), and since then, many studies have reported these effects that are influenced by traffic rush hours (e.g., Cerro et al. 2014; Henschel et al. 2015), population size (e.g., Butenhoff et al. 2015) and degree of urbanisation (e.g., Huryn and Gough 2014). The phenomenon can also be seen in China; for example, both Tang et al. (2008) and Lei et al. (2015) reported lower concentrations of primary pollutants at weekends compared with those on weekdays in Shanghai and Beijing, respectively.

Holidays often mean that factories are close and people spend more time at home, leading to lower pollutant concentration, which has been termed the holiday effect. For example, the drastic decrease of primary pollutant emissions (83–98% of NO) during the Jewish Day of Atonement was reported by Levy (2013). Meanwhile, Tan et al. (2009) revealed lower concentrations of NOx, CO and non-methane hydrocarbons at Chinese New Year. As this is such a major holiday, it is hardly surprising that the holiday effect is widely observed. The concentrations of SO_2_ and PM10 in Taiwan from 1994 to 2006 were lower during Chinese New Year (or the Spring Festival, sometimes termed the Lantern Festival in Taiwan), while the concentration of ozone was higher (no titration by NO). In China, the Spring Festival led to a general improvement in air quality (e.g. Li et al. 2006; Tan et al. 2009; Gong et al. 2014; Lei et al. 2015; Zhao et al. 2015). However, this is not always true as research on the Spring Festival effect in 31 key Chinese cities found no significant reduction in the concentration of PM2.5 and PM10 during the holiday compared with the period that followed (Chen et al. 2014). In Shanghai, there can also be a Spring Festival rush hour as people prepare for the holiday (Huang et al. 2012), which not only enhances primary pollutant concentrations but also showed sharp peaks in particulate peaks during past New Year celebrations (Zhang et al. 2010).

A range of cultural activities can directly enhance primary pollutants, such as the Sunday roast of Victorian England (Brimblecombe 1987) or barbecues (Tsai et al. 2015). There are numerous reports on the impact of key events where fireworks contribute to visibility reduction (Singh et al. 2015), toxic metals (e.g., Camilleri and Vella 2010; Kong et al. 2015a), illicit use of the metalloid arsenic (Sterba et al. 2013) and enhanced particulate loads in: Spain (Moreno et al. 2010), Slovenia (Mlakar et al. 2012), the USA on Independence Day (Seidel and Birnbaum 2015) or German New Year (Drewnick et al. 2006), in India during the Diwali festival (Ravindra et al. 2003; Perrino et al. 2011; Chatterjee et al. 2013; Bhatnagar and Dadhich 2015) or the numerous festivals that occur throughout the summer in Malta (Camilleri and Vella 2010). Air pollution from fireworks is frequently observed at the Chinese Lunar New Year (Spring Festival) period; widely celebrated in Asia and within the Chinese diaspora. Firecrackers are thought to drive away evil spirits, that lurk around to torment human beings, but the noise causes them to vanish into thin air (Wong 1967); however, such celebrations contribute to a reduction in air quality. Tsai et al. (2012) found that the average concentration of PM10 rose to high levels over Kaohsiung Harbour, influenced by fireworks during Taiwan’s lantern festival. Gong et al. (2014) reported a reduction in aerosol during the Spring Festival and revealed that the concentrations of major air pollutants had significantly decreased around the holiday, but a short-term peak could be seen for PM10, NO_2_ and SO_2_, due to fireworks along with peaks in the concentration of a range of metals Sr, K, Ba, Pb, Al, Mg, and Cu, (Chang et al. 2011), dicarboxylic acids (Wang et al. 2007). There has been concern that even relatively short exposures to metallic elements from fireworks might have health implications (Yang et al. 2014). Additionally perchlorates associated with fireworks as an oxidant can have health effects along with the potential to cause broader ecological damage (Sijimol and Mohan 2014). The impact of New Year fireworks on air quality has been studied in a number of Chinese urban areas: Yanshui in Taiwan (Chang et al. 2011), Beijing (Wang et al. 2007), Jinan (Yang et al. 2014), Lanzhou (Zhao et al. 2014), Nanning (Yan 2011), Pearl River Delta (Zhao et al. 2015), Wuxi (Cao 2014) and Xian (Wang et al. 2008; Shen et al. 2009; Zhou et al. 2013).

**Fig. 1:**
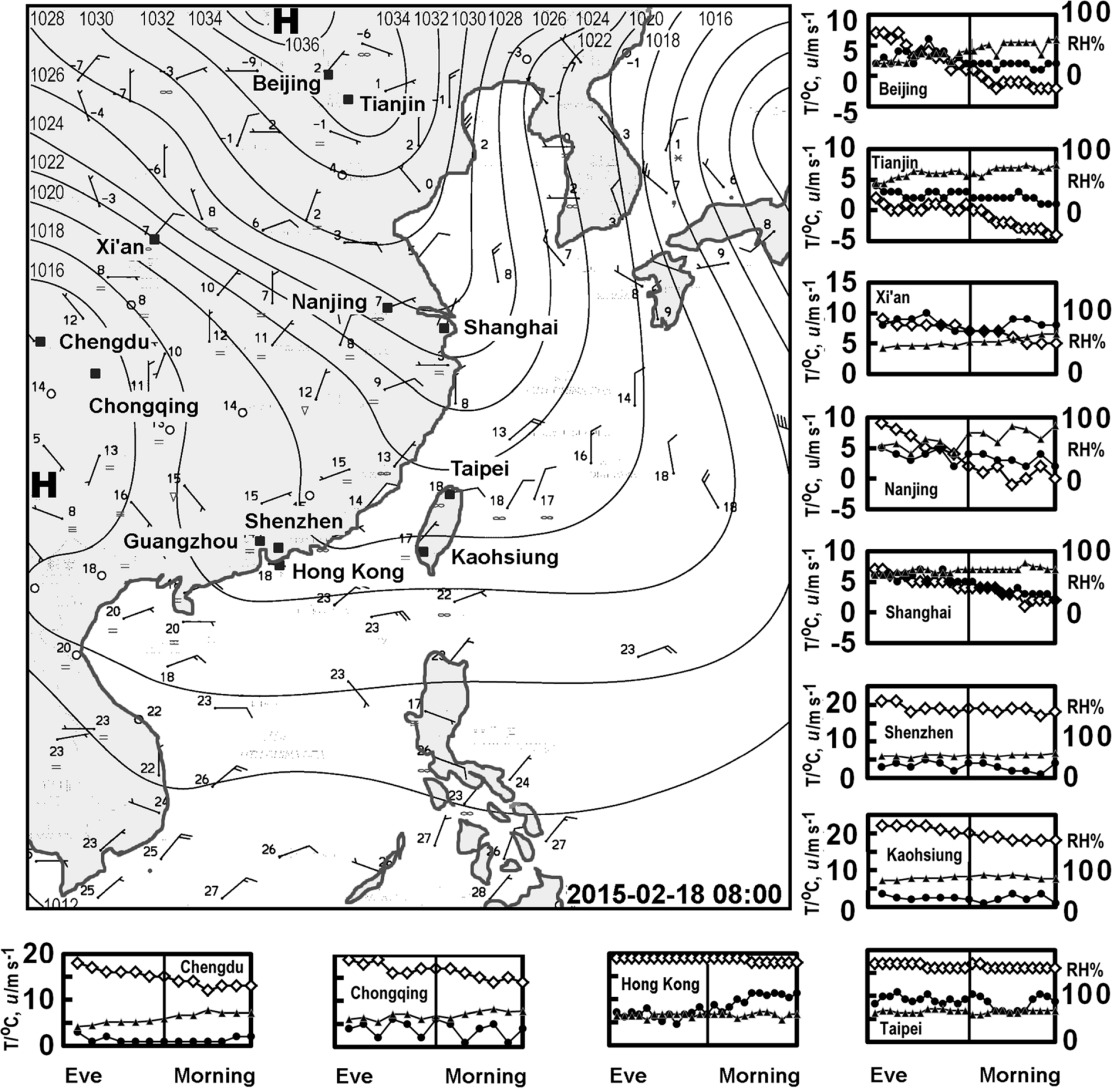
Meteorological chart of the region which includes the locations of cities discussed in the text, with the pressure field is adopted from (http://www.hko.gov.hk/wxinfo/currwx/wxcht.htm). The insets show the temperature (T, open diamonds), wind speed (*u*, dots) and relative humidity (RH, triangles) across arrival of the New Year from 18:00–06:00. Note: Guangzhou is not shown because of space limitations, but it is similar to Shenzhen, although the winds are slacker

This paper examines the cultural and administrative characteristics affecting the pattern of pollutants on the eve of Chinese New Year, 2015. Previous studies have examined Chinese New Year in specific locations, often describing the holiday effect. Here, we focus on the concentration of particulate matter derived from fireworks in cities across the region to examine the influence of differing approaches to regulation. The paper considers a number of Chinese cities (Beijing, Chengdu, Chongqing, Tianjin, Xi’an, Nanjing, Shanghai, Guangzhou and Shenzhen, along with Hong Kong (a Special Administrative Region) and Taipei and Kaohsiung (Taiwan). The cities are marked on the weather chart displayed in Fig. 1. We review the impact of this celebration across Greater China and see 2015 as an exemplar of the types of changes that may be underway. Evidence of aerosols from fireworks were found from aerosol characteristics in Xinxiang (Feng et al. 2016) and Tianjin (Liu et al. 2016) during the celebrations of 2015. It also places earlier work into perspective and allows us to consider of the role of regulatory activities in the face of the social norms of China’s *culture of Nian* when mythology justifies the use of fireworks to frighten evil spirits (Chao et al. 2014; Ye et al. 2016). Regulatory activities that restrict the use of fireworks have been evident in China since the 1990s, but in 2006, Beijing initiated a policy to allow people to use fireworks over limited periods although the application of such restrictions on fireworks may vary over time. It is often believed that it may be hard to gain broad acceptance of tough regulation regarding the use of fireworks at New Year. Despite this, there are some cities that seem to have grappled with the problem quite successfully for a considerable period of time. Hong Kong, possibly due to its colonial past, has had strict regulations regarding fireworks, while nearby Guangzhou and Shenzhen have tended to retain bans introduced in the 1990s. The paper will contribute to the debate on whether enhanced regulatory activity can be undertaken in a sensitive way, which is successful in retaining cultural values.

## Method

The date of Chinese New Year is set according to the lunar calendar. In 2015, the first day of the New Year was 19 February. Since the holiday period is different in different locations, our definition of the holiday period follows the local statutory definitions:

- Mainland China: Starting 18 February, ending 24 February.
- Hong Kong: Starting 19 February, ending 21 February
- Taiwan: Starting 18 February, ending 23 February.

In this study, the hourly and daily measurements of six pollutants were taken as PM2.5, PM10, CO, NO_2_, O_3_ and SO_2_ concentrations from available air quality monitoring stations in each of the cities for the 2015 New Year period. In much of the analysis, only the particulate matter was used, as the particulate matter concentrations show the clearest signature of the New Year event. The data were derived from observations made by various government agencies, which provided information as shown below:

- Mainland China: Data for 11 monitoring stations in Beijing and Shenzhen, ten in Guangzhou and Shanghai, eight stations in Tianjin, seven stations in Chengdu, 12 stations in Chongqing, 13 stations in Xi’an and nine stations in Nanjing (http://aqicn.org/city/).
- Hong Kong: Hong Kong Environmental Protection Department (HKEPD) data for 12 general stations and three roadside stations (http://epic.epd.gov.hk/EPICDI/air/station/?lang=en)
- Taiwan: Taiwan Environmental Protection Administration (TEPA) data for seven monitoring stations in Taipei and 12 stations in Kaohsiung (http://taqm.epa.gov.tw/taqm/en/HourlyData.aspx).

Meteorological data was taken from *Weather Underground*: https://www.wunderground.com/history/ sites in some cities needed to be stratified into various administrative areas, and although these are somewhat arbitrary, this has been done as follows: (i) Beijing; the inner city was defined as urban area i.e. inside the 2nd Ring Road, with suburban being the area between 2nd and 5th Ring Road. Outskirts are beyond 5th Ring Road, with rural the furthest sites or outer suburbs (https://en.wikipedia.org/wiki/Beijing). (ii) Tianjin; the Heping District is defined as urban while the peripheral administrative divisions a denoted as suburban (https://en.wikipedia.org/wiki/Tianjin). (iii) Shenzhen; Luohu, Futian and NanShan are defined as urban areas. Guangming and Dapeng are the new districts and remote, so classified as rural areas, with other districts are defined as suburban (see: https://en.wikipedia.org/wiki/Shenzhen).

The correlation between particulate concentrations and meteorological variables was determined using *Wessa Free Statistics Software* (https://www.wessa.net/), which was convenient as it gave Kendall *τ* and Spearman *r*_s_ in addition to the parametric Pearson *r*. The short data sets from individual sites in cities were compared using the Wilcoxon signed-ranks test. This was used most typically to compare concentrations measured during the celebrations [00:00–02:00] with those before and afterwards ([22:00–24:00] and [02:00–04:00]). This test was adopted as an alternative to the paired *t* test when assessing the difference between measurements from the monitoring stations across given cities, because the concentrations seemed skewed. The results of this signed-rank test are reported with the significance as a two-tailed *p* value, the test statistic *W* and the number of pairs *n* (i.e. sites). As the number of sites in the cities was small (i.e. 7–13), the observed value of *W* must be referred to an exact sampling distribution (Mosteller et al. 1973). In this work we used the on-line calculators available at the *VassarStats Website for Statistical Computation* (http://www.vassarstats.net/).

## Results

In addition to showing the position of the cities considered in this study, Fig. 1 also gives an indication of the overall weather conditions. It suggests a colder north, with a high-pressure area that probably led to stable conditions and the chance to build up pollutants. In the north of China, high pollutant concentrations can result from the large amounts of fuel required for heating. In the warmer south dispersion along the coast means, air pollution tends to be lower in the winter season. The annual particulate concentrations in Beijing, Tianjin, Xi’an are typically the highest. The cities of the coastal south are usually lower, along with Hong Kong and Taiwan. Measurements of temperature, wind speed and relative humidity from each of the cities are shown in the small graphs that surround the meteorological chart. Much as would be expected, these do not suggest sharp changes with the transition to the New Year.

**Fig. 2:**
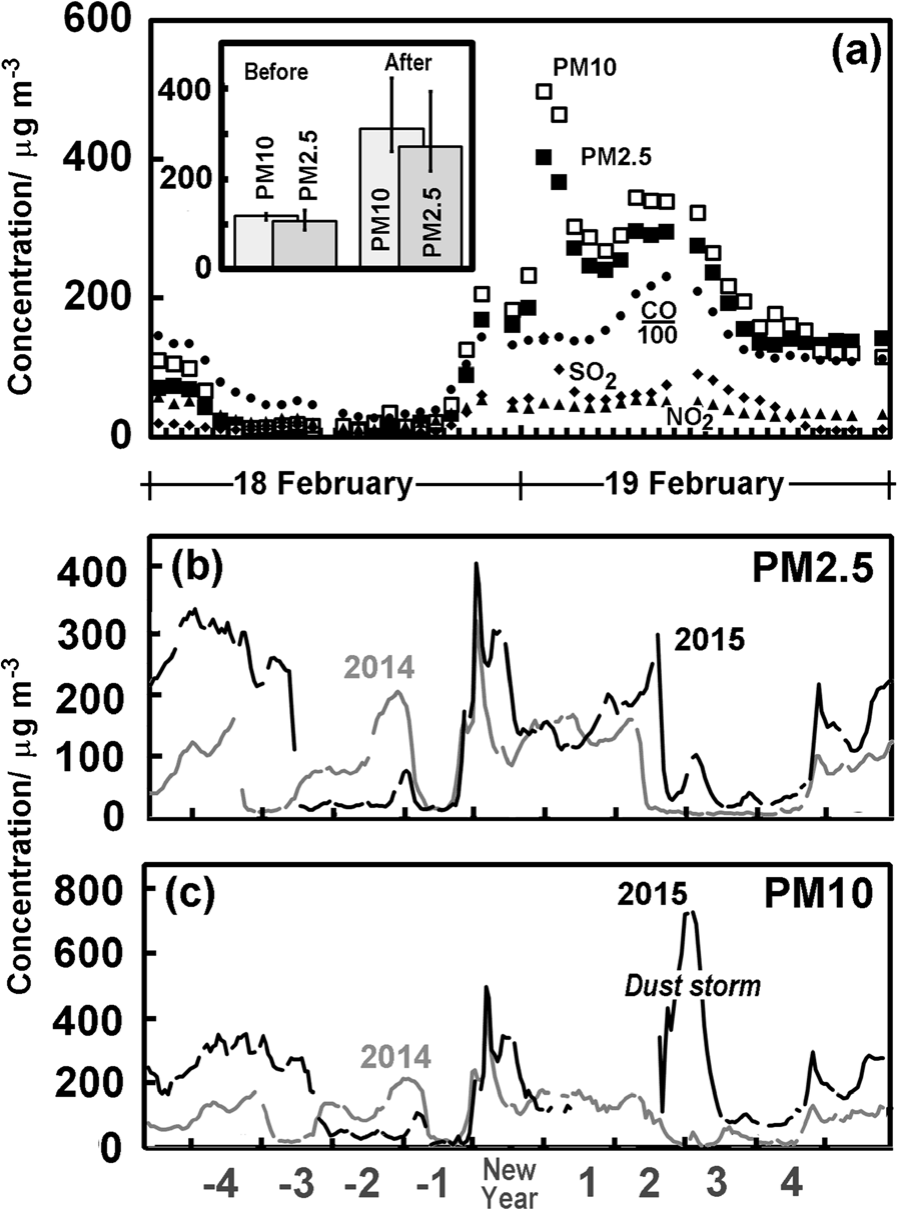
New Year air pollutant concentrations in Beijing. **a** Average pollutant concentrations in Beijing around the period of Chinese New Year. Open squares denote PM10 and closed squares PM2.5, while smaller dots diamonds and triangles denote CO, SO_2_ and NO_2_. Note: CO measurements have been divided by a hundred to bring them to a useful scale. Inset: comparison of the median of New Year’s Eve (18:00–24:00) PM10 (lighter shaded bars) and PM2.5 concentrations (darker shaded bars) and those early morning of the New Year (00:00–06:00) in Beijing. (b) Hourly PM2.5 concentrations averaged across Beijing for days around Chinese New Year for both 2015 (black line) and 2014 (grey line). (c) Hourly PM10 concentrations averaged across Beijing for days around Chinese New Year for both 2015 (black line) and 2014 (grey line) Note: The peak on the third and fourth day after New Year is associated with a dust storm referred to in the text

Figure 2a shows the average pollutant concentrations for Beijing on New Year’s Eve and New Year’s Day of 2015. We can see that there is a dramatic change in the concentration of particles just after midnight. It is also associated with an increase in SO_2_ as observed elsewhere (Yan 2011; Chatterjee et al. 2013) and hardly unexpected given the level of sulphur in fireworks. However, there is no substantial change in CO which would be expected from traffic, in line with Li et al. (2006). Neither do we see a sharp rise in NO_2_, in the early morning, but there are some increases through New Year’s Day, probably from traffic. The changes in particulate matter are so clear in the temporal plots, that the concentrations of PM10 and PM2.5 are used here as a marker of pollution derived from fireworks, and we restrict our study to these rather as other markers such as percholorate and the metals thought distinctive they are not available for a large number of sites. The differences in particulate concentrations in Beijing between New Year’s Eve [18:00–24:00] and the morning of the New Year [00:00–06:00] are shown in the inset in Fig. 2. These are displayed as the median PM2.5 (darker shaded bars) and PM10 (lighter shaded bars), where the upper and lower quartiles are plotted as “error bars”. The Wilcoxon test revealed significant differences at individual stations across the city before and after midnight for both PM2.5 (median 107 μg m^−3^ [18:00–24:00]; median 275 μg m^−3^ [00:00–06:00] *p*_2_ = 0.0036, *n* = 11) and PM10 (median 120 μg m^−3^ [18:00–24:00]; median 310 μg m^−3^ [00:00–06:00] (*p*_2_ = 0.005, *n* = 8). The HYSPLIT (Stein et al. 2015) back trajectory at this time suggests air from an area to the north on the previous day. This includes the non-urbanised Yan Mountains and the grasslands of Inner Mongolia beyond, which make long-range transport an unlikely source of the very high peaks in particulate concentrations.

Figure 2b, c show the hourly particulate concentrations averaged across Beijing for the days around Chinese New Year for both 2015 (black line) and 2014 (grey line). The temporal changes in Beijing over these longer periods before and after the Chinese New Year reveal no further obvious sharp peaks. However, a dust storm occurred in late February 2015 (RV 2015) and is evident 3 days after New Year, when a broad area of dust crossed Beijing driven by a northwest airflow in Mongolia. This led to more than 10 h of high PM10 concentrations, which peaked around 714 μg m^−3^ (Cheng et al. 2015).

**Fig. 3:**
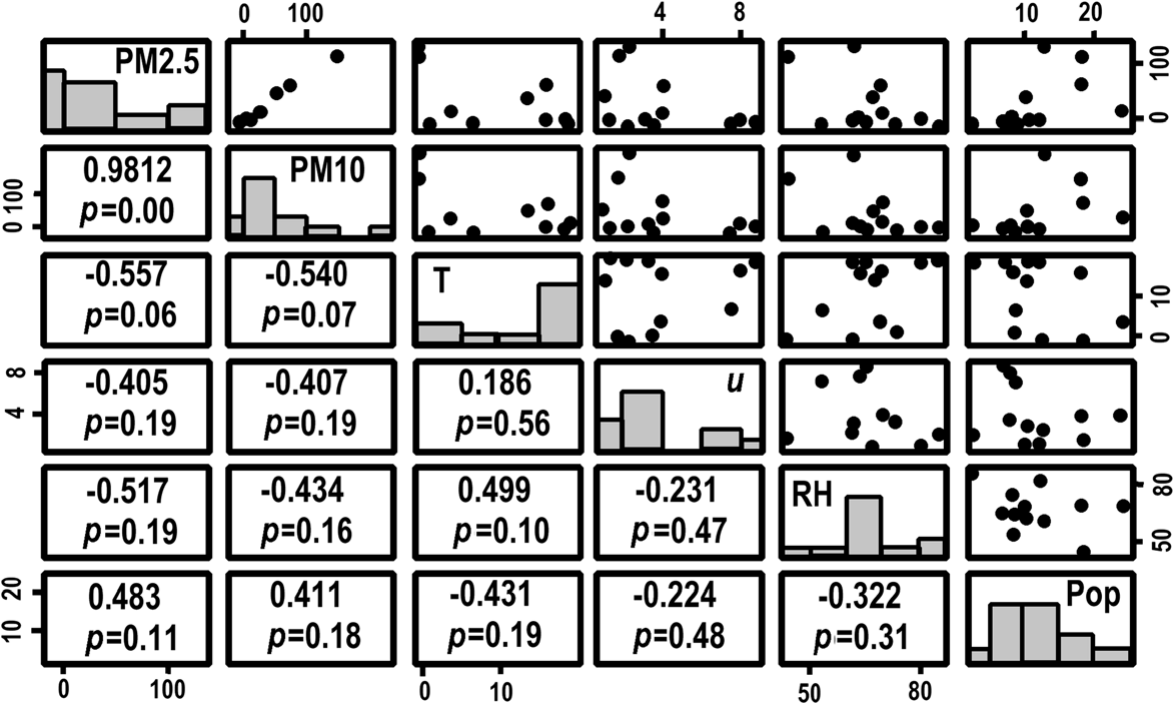
Correlation matrix between the additional PM2.5 concentration, additional PM10 concentration, temperature (T), wind speed (*u*), relative humidity (RH) and urban population (Pop) measured across the period 00:00–03:00. The correlation coefficient and *p* values are given in the lower left hand part of the matrix, while bivariate plots are shown in the upper right, with each large dot representing a city. Here the units are PM2.5 and PM10 as microgarm per cubic meter, T as degree Celsius, wind speed in meter per second, RH as % and population in millions. The histograms on the diagonal show the distribution of data for each variable

It is important when comparing cities across Greater China that we determined that any effects that changes in particulate load could be attributed to fireworks and not simply derived from different meteorological conditions. The potential for high concentrations observed on the arrival of the New Year to be related to temperature, wind speed and relative humidity in addition to urban population was determined by correlating measurements of average PM2.5 and PM10 concentrations 00:00–03:00 (four measurements) across a city with the meteorological parameters over the same period. The intensity of pollution at New Year was additionally estimated by subtracting the average of three measurements immediately before and after the New Year peak to get a sense of potential additional particulate matter from fireworks. It is these peaks in pollution that likely derive from the additional contributions from New Year fireworks that were correlated in the graphs and correlation matrix shown in Fig. 3. This figure shows the Pearson correlation coefficient, but the non-parametric Kendall *τ* and Spearman *r*_s_ (not shown) were little different.

The graph of the relationship between the PM2.5 against PM10 is the only one in Fig. 3 that is particularly convincing; all the others being rather scattered. Each dot in the plots represents a city. The correlation matrix in the lower left of Fig. 3 suggests that there is a significant relationship between PM2.5 against PM10 (*r* = 0.981 and *p* = 0.00). This is also true for correlation between the directly observed PM2.5 and PM10. (not shown but *r* = 0.961 and *p* = 0.00). There is a slight though weaker negative correlation between added PM2.5 and PM10 and temperature (PM2.5: *r* = −0.557, *p* = 0.06 and PM10: *r* = −0.540 *p* = 0.07). This seems likely to arise because pollution from fireworks is rather high in the two colder northern cities (Beijing and Tianjin), which have high peaks in particulate concentrations in the early hours of Chinese New Year. Not surprisingly, wind speeds also seem negatively correlated with peaks in PM2.5 against PM10 (shown in Fig. 3) and measured particulate matter concentrations (not shown); albeit none are statistically significant. Population shows a slight positive correlation with particulate matter, but not at significance levels (> 90%). The generally low level of significance apparent from the correlation matrix leaves us with an impression that the New Year peaks in particulate concentrations are not predominantly driven by meteorological conditions. This adds to the evidence presented in Fig. 1, which suggests no sharp changes in meteorological conditions at the beginning of the New Year.

**Fig. 4:**
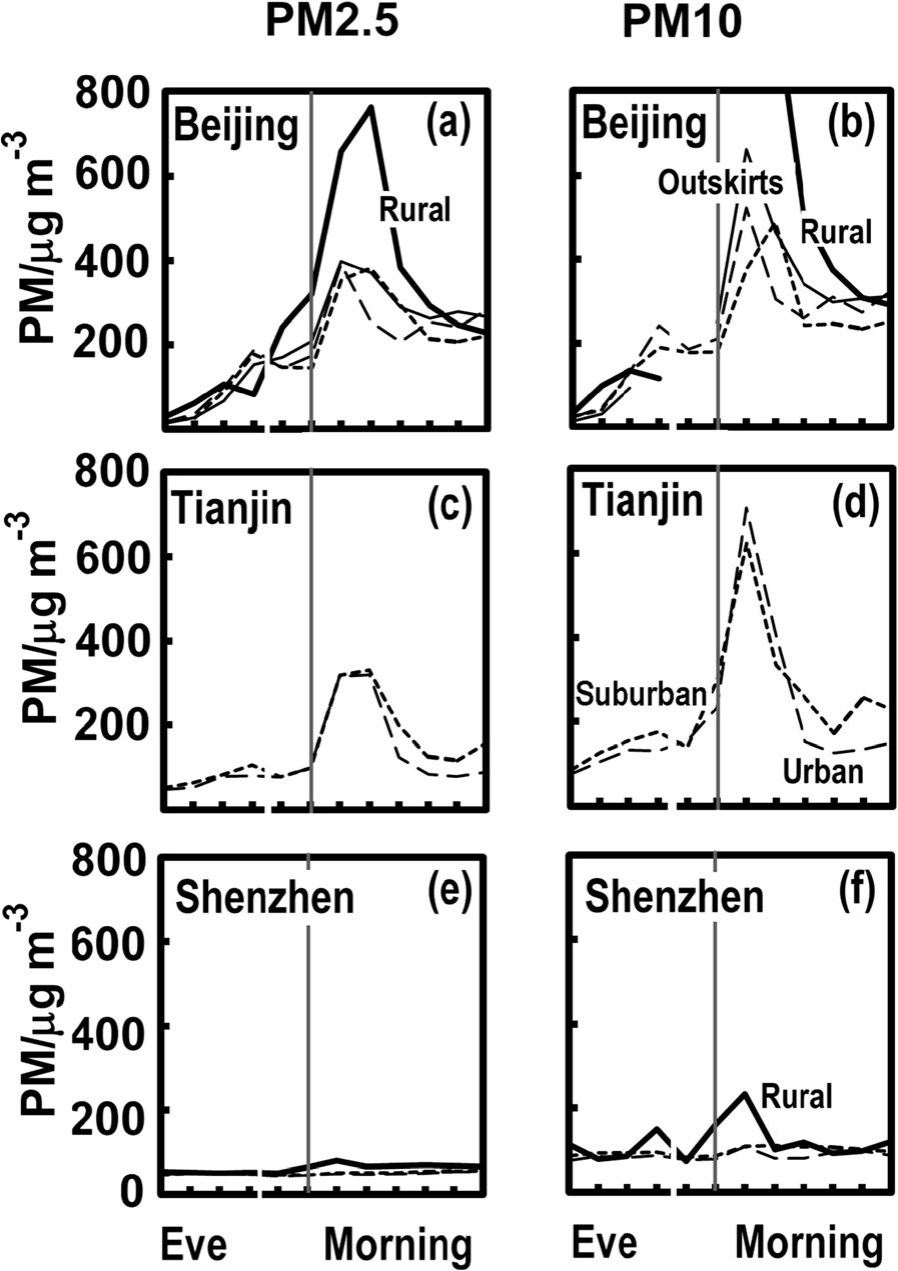
PM2.5 and PM10 concentrations on the night of the New Year 2015 (18:00–06:00) in Beijing (**a**, **b**), Tianjin (**c**, **d**) and Shenzhen (**e**, **f**). The lines show trends in urban (long dashes), suburban (short dashes), outskirts (fine) and rural (bold) areas. Note: The data from Mainland China have no measurements in the record at 22:00 (i.e. [21:00–22:00]), which is illustrated by a gap in the individual plots

The particulate matter observations explored in Fig. 3 do not consider the measurements across each city. There are so many stations, so here, PM2.5 and PM10 concentrations from the night of New Year are averaged for various classes of sites: urban, suburban, outskirts and rural (defined earlier) for Beijing, Tianjin and Shenzhen as shown in Fig. 4a–f. It is noticeable that in both Beijing and Shenzhen especially, the outskirts of the city or rural areas may have the most distinct peaks in particulate concentrations as the New Year begins.

**Fig. 5:**
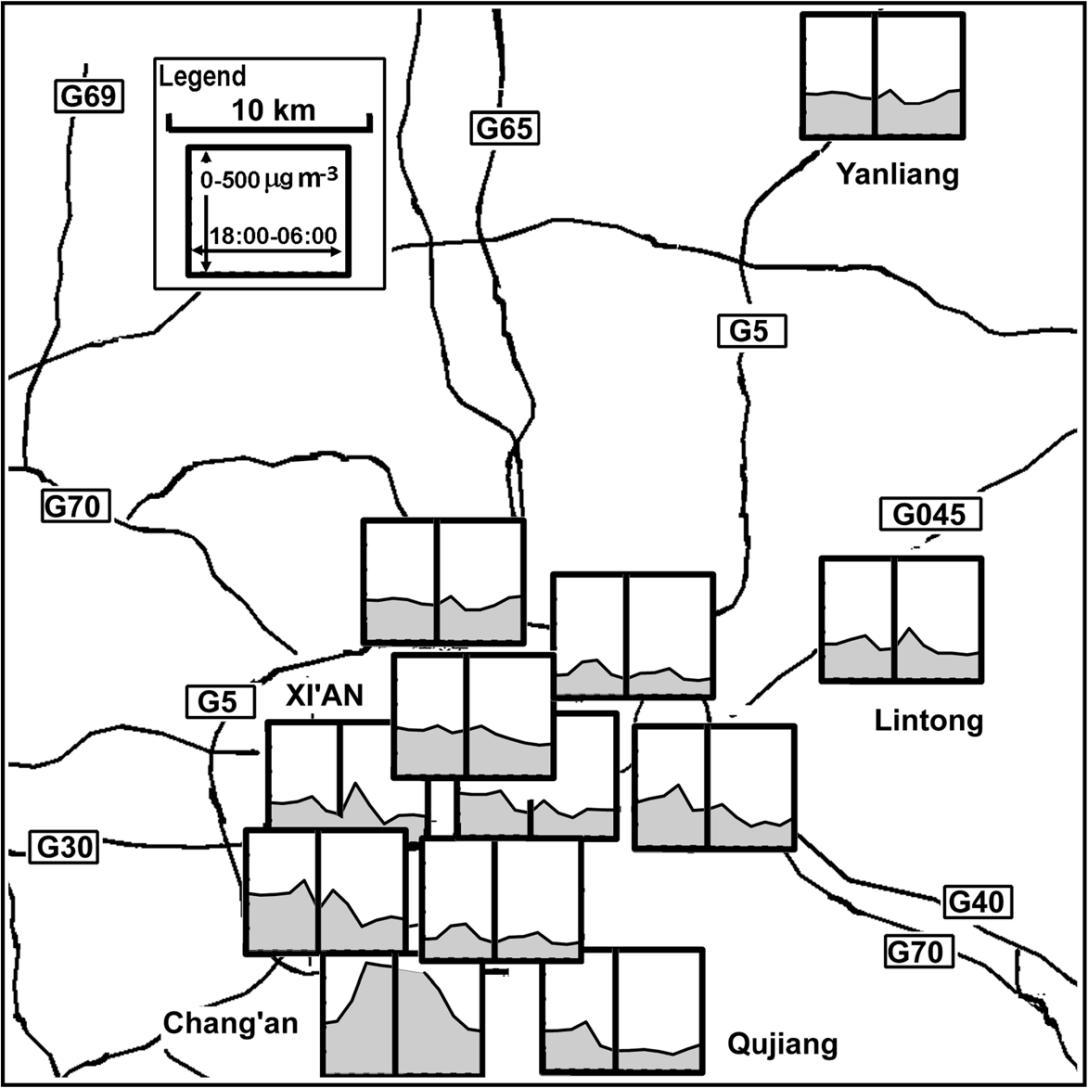
The PM10 concentrations at individual stations in Xi’an on the night of the New Year’s Eve (18:00–24:00) and the following morning (00:00–06:00) plotted as a map. The dark vertical line denotes midnight and the number designating of major roads are enclosed in rectangles

Slightly more detail is mapped for PM10 from individual sites from Xi’an across New Year’s Eve and the following morning in Fig. 5. The small outlying town of Lintong (the district has 0.66 million people, but the town is only ~ 75,000) and Yanliang District of Xi’an (district population ~ 28,000) show only modest, though clear, impact from celebrations. Obviously, the New Year is welcomed with fireworks in the early morning, but the population is low so the impact probably remains small, with just a slight increase of PM10 concentrations in the early morning. Qujiang in the south is a residential area popular with visitors and shows little variation through the evening, while the increased pollution throughout the evening at Chang’an may be due to a combination of factors, as the area is associated with both residential occupancy and industrial enterprises. The district covers a large area, and although the population density is low, it has more than 600 villages, with the largest number in the areas that surround Xi’an (XEN 2011; SYCCA 2011), which may account for the broad peak and high concentrations of PM10 over the New Year. The issue of the differences in firework peaks across the city and shifts in temporal behaviour aligns with the earlier observations of Wang et al. (2008). They argue that the average concentration of PM10 and SO_2_ in Xi’an peaks at around 01:00 on New Year’s Eve and that the measurements from stations around the city differ based on population density, firework discharge etc., with the PM10 increase tending to be highest in suburban areas (Wang et al. 2008).

Cities across China have adopted rather different regulations with respect to the use of fireworks at New Year. As noted by Li et al. (2006), who investigated air pollution in Beijing during the spring festival over the period 2000~2006, the intense use of fireworks over a short time created a serious air pollution problem. There had been attempts at controlling the problem since the 1990s, but from 2006, Beijing initiated a policy that that allowed people to celebrate with fireworks, but restricting use from Chinese New Year’s Eve to 24:00 the following day for locations within the 5th Ring Road. There was evidence of high pollutant concentrations across the city, although this improved with the adoption of regulations (Li et al. 2006). Despite the attempt to alleviate high pollution levels through time restrictions on the use of fireworks (Appendix Table 1), there is still much evidence of large concentrations of air pollutants, most notably particulate matter and sulphur dioxide across Beijing (as shown in Fig. 2).

**Fig. 6:**
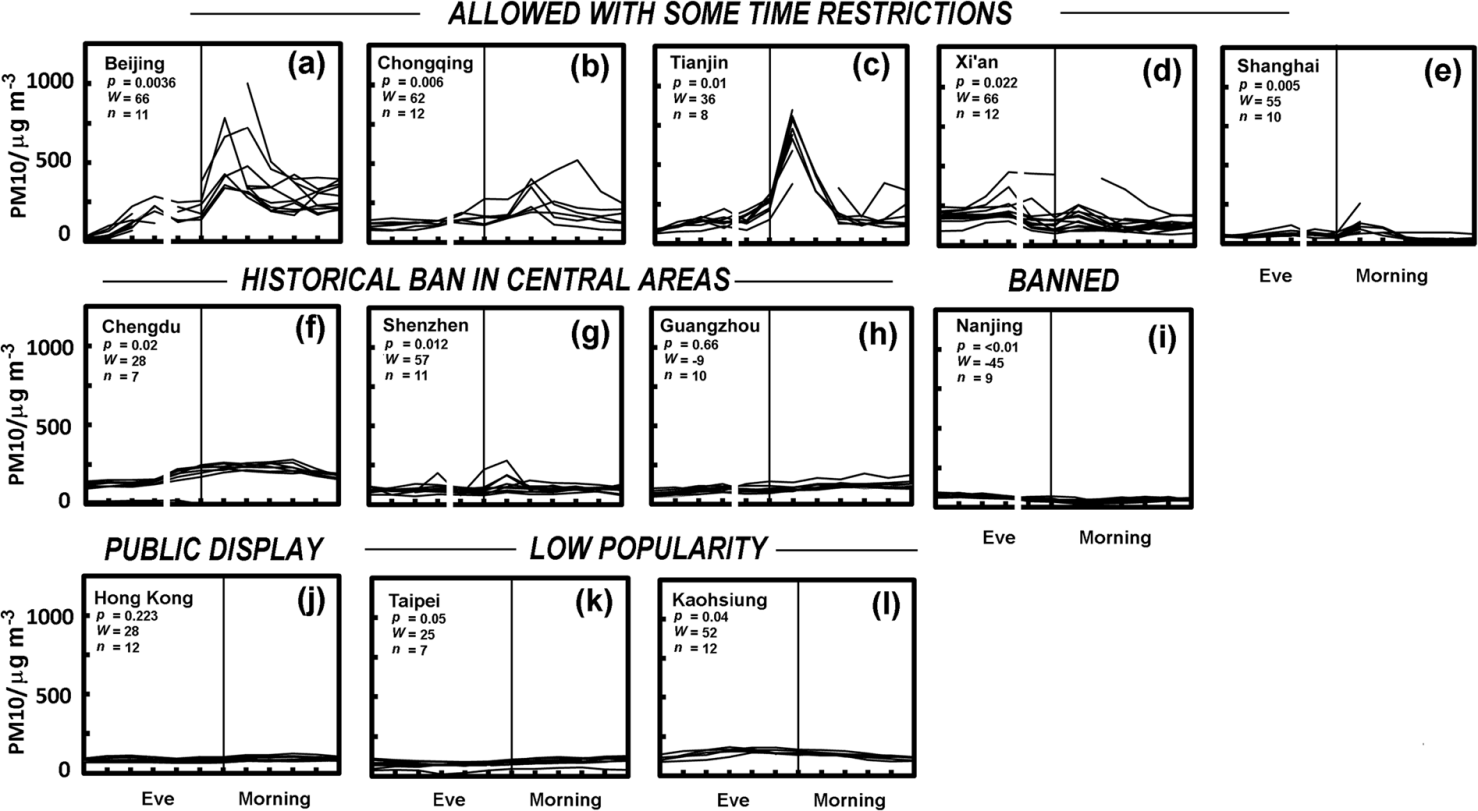
Trends in PM10 concentrations at individual stations in various urban areas on New Year’s Eve and the following morning. This includes stations in cities that allow fireworks, although with time restrictions in some cases: **a** Beijing, **b** Chongqing, **c** Tianjin or **d** Xi’an, **e** Shanghai; those with historical restrictions in central areas: **f** Chengdu, **g** Shenzhen and **h** Guangzhou or a complete ban **i** Nanjing and those of **j** Hong Kong, **k** Taipei and **l** Kaohsiung. Notes: tick marks on the x-axis denote hours and the small break and shift in the midnight line arises because the 22:00 values are missing from records for Mainland China. The text on each shows the two-tailed *p* value, the test statistic *W* and the number of sites *n*, which account for the difference at individual sites between measurements during the celebrations [00:00–02:00] and those before and afterwards ([22:00–24:00] and [02:00–04:00]).

Observations made across the hours immediately before and after New Year 2015 in sites from Mainland China, Hong Kong and Taiwan are shown in Fig. 6. The Wilcoxon signed-ranks test was used to test the difference at individual stations in given cities during the early morning peak [00:00–02:00] and the hours before and after ([22:00–24:00] and [02:00–05:00]), with results tabulated on the panes of the figure.

The profile of changing PM10 concentrations at individual stations in Beijing for New Year’s Eve and the early morning of the New Year is shown in Fig. 6a. It is clear that there is a widespread increase in the concentration of particulate matter across the city in the early morning and a high significance revealed by the Wilcoxon test (*p* = 0.0036, as tabulated in Fig. 6a). As noted earlier, the changes are largest at stations remote from the city centre. Examining individual station records suggests that Huairouzhen which lies beyond the 6th Ring Road has concentrations that appear to exceed 1000 μg m^−3^. Concentrations are also high, in excess of 500 μg m^−3^, in two other locations: (i) Wanshouxigong in the Baizhifang Residential District in the southwest just inside the 2nd Ring Road and (ii) Shunyixincheng in the Shunyi District northeast of Beijing’s Capital International Airport. Thus, the very highest concentrations are often away from the centre of the city; early morning peaks are observed at all sites around the city. Here, concentrations seem to be some 250 μg m^−3^ above what might be expected where there were no contributions from fireworks in the concentrations measured in the early hours of the New Year (see the trend lines of Fig. 6a). The attempt to regulate the times permissible for fireworks seems ineffective in keeping particulate concentrations low. However, Beijing has started to include public displays at around midnight on New Year’s Eve. High air concentrations during the celebrations of 2017 drew considerable comment, which may increase enthusiasm for such public displays.

Chongqing (Fig. 6b) and Tianjin (Fig. 6c) have somewhat similar policies to Beijing, restricting the time over which fireworks are permitted in the central parts of the city (see Appendix Table 1). All sites show very pronounced peaks in PM10 concentrations in the early morning in Tianjin. Xi’an (Fig. 6d) has no fireworks ban for Chinese New Year although typically fireworks should not be used. As seen in Fig. 5, measurements from of Xi’an show more variable profiles in PM10 concentrations through the night, which may explain the low statistical significance of these changes. Nevertheless, a peak in the PM10 concentrations is evident in the early morning for a number of sites, but the concentrations are not especially high when compared to Beijing, Chongqing and Tianjin, but Xi’an is a smaller city. The changes in Shanghai (Fig. 6e) are rather small, with peaks suggesting the addition of only 50–100 μg m^−3^ to early morning concentrations of PM10, nevertheless, the early morning increase across the city is significant (*p* = 0.005).

The cities of Chengdu and Shenzhen have bans in certain regions (Appendix Table 1). Chengdu (Fig. 6f) shows little evidence of a sharp morning peak in particulate concentrations suggesting some success with their regulation of pollution from fireworks. There was a general increase in pollution in this city on New Year’s Day (*p* = 0.02), but the early morning changes are small, on average only 15 μg m^−3^. Figure 6f makes a less convincing case for a firework source in Chengdu compared with cities such as Beijing. In Shenzhen (Fig. 6g), it seems that long standing regulations are fairly effective suggesting little increase in PM10 concentrations, although in the city, overall the peak in the early morning hours is significant (*p* = 0.0012). However, this arises from clear peaks in particulate concentrations (Fig. 6g) associated with measurements made in: Kuiyong, Nanyou, Nan’au and perhaps less clearly Xixiang. These sites are all rather distant from the city centre, where controls are in force. Once these remote sites are removed, the significance of Shenzhen’s early morning increase in PM10 declines (i.e. *p* > 0.05). With the exception of these remote sites, the impact of fireworks in Shenzhen appears relatively small compared with changes seen in Beijing and Tianjin. The main city of Guangdong Province, Guangzhou has permitted fireworks in suburban areas, although it has banned their use in central urban areas since 1992. Here, results suggest little clear contribution from fireworks, with early morning values or PM10 being somewhat lower than other times (as shown by the negative *W* statistic in Fig. 6g). Nanjing introduced strict controls in 2015 and shows no evidence of a firework contribution to particulate matter (Fig. 6i) and a significantly negative *W* statistic.

Outside the Chinese Mainland, Hong Kong (Fig. 6j) and the cities of Taiwan: Taipei (Fig. 6k) and Kaohsiung (Fig. 6l), seem to reveal little evidence of pollutants from fireworks. They perhaps hint at an effective reduction of emissions. In Taiwan fireworks are allowed, although not necessarily used to a great extent or especially popular. There may be some increases in early morning PM10 in Taipei and Kaohsiung (*p* = 0.05, *p* = 0.04), but they remain small (Fig. 6k, l). Temple parades may still use them although these are not primarily New Year’s Eve activities. The use of fireworks is spread throughout the year, with just a few temples celebrating a midnight event at the New Year (e.g. the God of War Temple in Yanshui, Tainan City). A particular focus in Taiwan has been the creation of a less polluting lantern festival, which is popular with tourists. The lanterns are often electrical, thus reducing the potential for air pollution (see TB 2016).

**Fig. 7:**
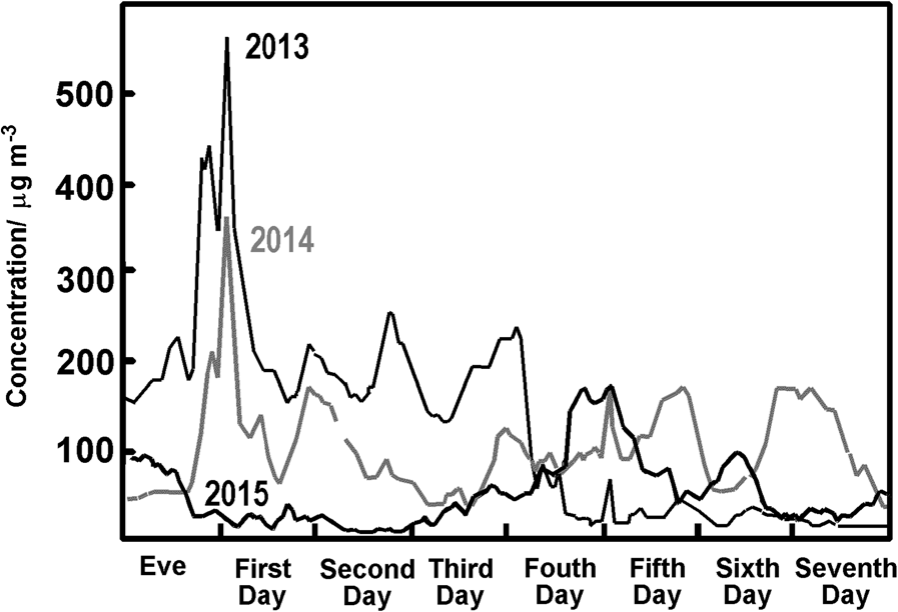
Average PM2.5 concentrations in Nanjing for New Year’s Eve and the week that follows for the years 2013 (black), 2014 (grey) and 2015 (bold black)

Looking at some cities in more detail, we turn first to Nanjing, which had problems in previous years (Kong et al. 2015b). Its government carefully examined the impact of fireworks on the city and determined that over the years 2010, 2011and 2012, some 80, 195 and 170 t of firework debris accumulated each day during the Chinese New Year holidays (NJHB 2015), although after New Year’s Eve, the amount would be much higher. In 2013, the government estimated that fireworks waste involved a collection of 2140 t of debris following the celebrations. Such considerations led to the stricter control of fireworks in Nanjing, where improved air quality (Fig. 7) hints at the effectiveness of the recent ban. This provides evidence to a local government which has been anxious to show signs of improvement following the tougher regulation (NJHB 2015).

Across a city, there may be variations because of local regulations, as seen in Shenzhen (Fig. 6g), but significant spatial variation can be seen in some cities such as Xi’an (Figs. 5 and 6d). Here, some sites show peaks after midnight, some show peaks at other times during the evening, while others show little change.

## Discussion

Interest in restricting the use of fireworks in China spans more than two decades. This confronts some long-held cultural beliefs and deeply embedded traditions. Li et al. (2006) believed that regulations played an important role in reducing PM10 in areas of Beijing where fireworks were forbidden. Nevertheless, Wang et al. (2008) found a trend of increasing PM10 concentrations through New Year’s Eve in Xi’an in both urban and suburban areas, so concluded that the government should put forward a policy to ban fireworks. There has been a fairly restrictive ban on fireworks in the main areas of cities of the Pearl River Delta (Guangzhou and Shenzhen) since the early 1990s (Zhao et al. 2015). However, it has been argued that an increasing amount of PM2.5 on New Year’s Eve can still be observed in suburban areas in these two cities.

Overall our results suggest that while in some cities the celebration of New Year brings a noticeable decline in air quality, but a number of other cities seem to have made satisfying achievements in reducing the extent of the problem. The effectiveness of regulation might be expected in Hong Kong and Taiwan, given their different approaches to governance. Nevertheless, some cities on the Mainland: Chengdu, Shenzhen and Guangzhou, and most recently Nanjing seem to have relatively good air quality during the festivities. While a Nian culture still survives, it appears that regulation may be possible.

The evidence of distinctive peaks in air pollution from fireworks across individual cities can be variable, with the largest effects found at the outskirts, perhaps suggesting that regulations may be more relaxed at the margins. In cities where regulations are enforced, the evidence of fireworks activity in the PM10 concentrations seems to occur well away from the centre, such as in the more remote parts of Shenzhen, again evidence of lower concern well away from urban centres. Such locations might well benefit from considering the work of Mlakar et al. (2012) from Slovenia who make an argument for selecting areas for fireworks displays according to knowledge about air pollution dispersion. Although the focus here is on China, it suggests the relevance of regulation for other parts of the world where fireworks are used at specific times such as US Independence Day, Hindu Diwali (Perrino et al. 2011; Chatterjee et al. 2013) and Guy Fawkes celebrations of England and some Commonwealth countries (Singh et al. 2015). The example from China may be less relevant to other places, such as Malta, where more than 80 individual villages will use fireworks for local festivals, often on different days (Camilleri and Vella 2010).

## Conclusion

The celebration of Chinese New Year is associated with loud noises, firecrackers, coloured lanterns and, in many cases, air pollution. The cities of Beijing, Chongqing, Tianjin and Shanghai, where fireworks are allowed albeit with time restrictions, show significant (*p* < 0.05) peaks in the early hours of the New Year and substantial increases in PM10 concentrations (> 25 μg m^−3^). Pollution, waste and safety issues have seen regulatory pressures to restrict the use of fireworks and change behaviour for a number of years. Nanjing is particularly notable for its high air quality during the 2015 New Year, much improved over earlier years. To some extent, regulations have been more successful at reducing an obvious peak in particulate concentrations in the south of China. It may be because traditions are different, or that traditions have changed. The differences may also reflect provincial differences in regulatory procedures that could come apparent in a large country.

The desire to have celebrations that have a lower impact on air quality may well be achieved without entirely removing the celebratory nature of this important event in the Chinese calendar. Taiwan has been notable in promoting the event as more about lanterns than fireworks. Hong Kong has been consistent in adopting carefully controlled public displays of fireworks and to restrict private use. They also to ensure that those used are sourced to minimise pollution, not only in the air but also the deposition of toxic materials to the environment. The Environmental Protection Department analysis argues that “the National Day Fireworks Display and the New Year’s Eve Pyrotechnic Show… last for a short duration involving high-altitude fireworks discharge above the sea.... This accounted for 0.1 per cent of Hong Kong's total emission of RSP in 2011” (HKSAR 2013). In the past, firework displays were more frequent over Hong Kong’s Victoria Harbour. Now there is a celebration of the city each evening with a stunningly orchestrated display of lights on the major tall buildings of Hong Kong Island combined with beams from moveable searchlights and lasers. Thus, there is almost no potential for enhanced particulate emissions. Environmental regulation routinely limits the freedom of choices available to individuals, but the celebration of Chinese New Year may still be widely enjoyed even when restrictions on the use of fireworks are enforced.

## Appendix

**Table 1:** Regulations regarding fireworks for cities discussed in this study. Note: the populations in column 1 are as millions and are for the year 2015 except for Chengdu, Guangzhou, Shenzhen and Hong Kong (2014) and Taipei and Kaohsiung (2016) and the values are for urban except Xi’an (sub-provincial city), Shanghai (municipality), Nanjing and Hong Kong (total)

City	Regulation	Reference
Beijing 18.5M	Since 2006, the Beijing Government limited firework use, but allows them within the fifth ring road (on the Chinese New Year’s Eve until midnight.	http://news.sina.com.cn/s/2005-09-09/15516904684s.shtml
Chongqin 18.38M	Government allows fireworks on the New Year’s Eve until 01:00 the following morning.	http://cq.bendibao.com/jieri/2015115/51818.shtm
Tianjin 12.78M	Tianjin fireworks safety management approach: people can use fireworks in main areas on the New Year’s Eve until 02:00 the following morning.	http://m.9tour.cn/info/info_29122.html
Xi’an 8.7M	No strict regulations that ban so they can be used from New Year’s Eve to the fifteen day of Chinese calendar.	http://xa.bendibao.com/news/201526/50241.shtm
Chengdu 10.15M	In 2015, government issued new regulations banning the use of fireworks in all main areas within three ring roads .	http://chuansong.me/n/1161145
Shanghai 24.15M	In some areas fireworks are prohibited, but still allowed in part of central areas..	http://sh.people.com.cn/n/2015/0209/c134768-23847311.html
Shenzhen 10.63M	Since 2014, Government issued a strict policy that bans people to burn fireworks in all main areas during CNY.	http://sz.ce.cn/sy/gd/201501/30/t20150130_2015202.shtml
Guangzhou 11.26M	Since 1992, the government has banned the use of fireworks during spring festival in central urban areas, but are permitted in suburban areas.	http://opinion.cntv.cn/2014/01/12/ARTI1389484934462287.shtml
Nanjing 8.23M	Since 2015, a strict policy bans the use of fireworks in most areas.	http://nj.bendibao.com/jieri/2015211/51532.shtm
Hong Kong 7.23M	Public displays of environmentally friendly fireworks well established	http://www.info.gov.hk/gia/general/201302/20/P201302200272.htm
Taipei 8.50	Normally 08:00–22:00 but waived for New Year’s Eve and the first day of the New Year.	http://bepo.ctitv.com.tw/2016/02/24151/
Kaohsiung 2.54	Kaohsiung, Normally 06:00–23:00, but waived for New Year’s Eve and the first day of the New Year	http://www.appledaily.com.tw/appledaily/article/headline/20110831/33634693/
